# Validation of the International Prognostic Index and Subsequent Revisions for Diffuse Large B-Cell Lymphoma in Patients From the Middle East and North Africa Region

**DOI:** 10.7759/cureus.9620

**Published:** 2020-08-09

**Authors:** Faisal Alamer, Ahmad Alamir, Abdulrahman Alqahtani, Abdulrahman M Alkabli, Homod Alshabib, Moussab Damlaj

**Affiliations:** 1 Oncology, King Abdulaziz Medical City, Riyadh, SAU; 2 Medicine, King Abdullah International Medical Research Center, Riyadh, SAU; 3 Medicine, King Saud Bin Abdulaziz University for Health Sciences, Riyadh, SAU

**Keywords:** duodenal diffuse large b-cell lymphoma, international prognostic index, middle east north africa region

## Abstract

Diffuse large B-cell lymphoma (DLBCL) is a heterogenous disease with a variable prognosis. The International Prognostic Index (IPI), revised-IPI (R-IPI), and National Comprehensive Cancer Network-IPI (NCCN-IPI) have been developed and validated to predict prognosis in DLBCL. However, patients from the Middle East and North Africa (MENA) region were under-represented in such scores, and it is unclear whether ethnic background contributes to different disease biology or response to therapy. Following due Institutional Board Review approval, DLBCL patients diagnosed from January 2010 until December 2015 from the MENA region were retrospectively reviewed. A total of 122 were identified and further analyzed. There were 74 males (61%), and the median age at diagnosis for the cohort was 64 years (range: 18-98 years), with a median follow-up duration of 32.9 months (range: 0.2-123.7 months). Estimates of three-year progression-free survival found a significant difference among risk groups using all three prognostic models but were more discriminating among the groups using NCCN-IPI and R-IPI vs. IPI (p = 0.019 and 0.014 vs. 0.039, respectively). For overall survival estimates at three years, the NCCN-IPI was the best model compared to R-IPI and IPI (p = 0.0013 vs. 0.05 and 0.04, respectively). In conclusion, we validated that the IPI and its subsequent iterations were predictive of outcome in DLBCL patients from the MENA region; however, the NCCN-IPI appeared the most prognostic. These results warrant further confirmation.

## Introduction

Diffuse large B-cell lymphoma (DLBCL) is a commonly diagnosed non-Hodgkin lymphoma (NHL), accounting for one-quarter of NHL cases [1]. Approximately, half of the patients afflicted with DLBCL can be cured when treated with chemo-immunotherapy approaches typically consisting of combinational chemotherapy combined with the CD20 monoclonal antibody rituximab [2]. In Saudi Arabia, NHL is the third most commonly diagnosed malignancy with an incidence rate of 6/100,000, with the most prevalent NHL subtype being DLBCL [3].

DLBCL remains a heterogeneous disease with differing presentations and clinical outcome. Therefore, the identification of prognostic tools is of paramount importance to aid physicians to more accurately risk-stratify their patients. The International Prognostic Index (IPI) was originally developed in order to assess the impact of pre-treatment factors on outcome. The identified factors included age > 60 years, lactate dehydrogenase level (LDH) above normal, Eastern Cooperative Oncology Group (ECOG) performance status > 2, stage III or IV disease, and the presence of more than one extra-nodal site [4]. Cumulative risks are tallied to estimate the risk status in one of four categories, low, low-intermediate, high-intermediate, or high-risk disease, with significantly differing overall survival (OS) and relapse-free survival (RFS) among the groups.

As the original IPI was derived from cohorts that have not received rituximab, subsequent efforts were undertaken to revise this score in contemporarily treated patients. Sehn et al. undertook such an effort and the reported revised IPI (R-IPI) identified three prognostic groups - very good, good, and poor - again with differing outcomes [5]. Finally, the National Comprehensive Cancer Network (NCCN) did further refinement of the score to include age and LDH as continuous variables and the location instead of the number of extra-nodal lesions [6]. Using the NCCN-IPI, patients were again classified into four groups similar to the original IPI, i.e., low, low-intermediate, high-intermediate, or high risk.

Importantly, patients from the Middle East and North Africa (MENA) region were under-represented in the derivation of such prognostic scores. This raises important questions on whether the IPI and subsequent versions can accurately predict prognosis in this ethnic population. For example, an effort to validate the original IPI in a Chinese population reported that the presence of extra-nodal disease was not an important element in the score to stratify patients [7]. The aim from of analysis is to validate the original IPI and its subsequent iterations in a Saudi cohort from the MENA region.

## Materials and methods

Study design and patients

This was a retrospective single-center study conducted at King Abdulaziz Medical City, Riyadh, Saudi Arabia, following Institutional Board Review approval. Patients ≥ 14 years of age with a diagnosis of DLBCL from January 2010 until December 2015 were identified and data were retrieved through a query of the institutional Oncology database. Diagnosis of DLBCL was per the World Health Organization 2016 Classification and based on a combination of compatible morphology, immunophenotype, and genetic profile [8]. Clinical and pathologic variables were retrospectively abstracted. Majority of patients were treated with combinational chemotherapy containing rituximab. Patients who died prior to receiving therapy were excluded.

Computation of the IPI and subsequent revised versions

The IPI was calculated based on the following variables as previously described, with one point being assigned to each: age 60 years and older, elevated LDH level, stage III-IV disease, ECOG score, and more than one extra-nodal site of disease [4]. The total score was tallied and patients were stratified into different risk groups as follows: low with a score of 0-1, low-intermediate with a score of 2, high-intermediate with a score of 3, and high with a score of 4-5.

The R-IPI was computed using the same variables as the original IPI but with only the following three distinct prognostic groups: very good with a score of 0, good with a score of 1-2, and poor with a score of 3-5 [5]. Finally, the NCCN-IPI was computed by using the same variables but with further refinement of age into groups (≤40, 41-60, 61-75, and >75), LDH (normal, ≤3x upper limit of normal, or >3x upper limit of normal), and specifying the location of extra-nodal sites to bone marrow, central nervous system, liver, gastrointestinal tract, or lung, whereas ECOG and stage remained the same. Using the NCCN-IPI, patients were again classified into four groups similar to the original IPI, i.e., low with a score of 0-1, low-intermediate with a score of 2-3, high-intermediate with a score of 4-5, or high risk with a score of 6-8 [6].

Definitions and statistical analysis

All variables were collected retrospectively with baseline patient, disease, and treatment characteristics reported as frequency, median, and/or percentages. Comparisons between variables were made using the Pearson and Wilcoxon/Kruskal-Wallis tests for categorical and continuous variables as appropriate. The Kaplan-Meier method was used to estimate progression-free survival (PFS), and OS and was reported as a percentage with log-rank test for group comparison. The definition of OS was the time from the diagnosis of DLBCL until the date of death due to any factor or last documented follow-up. Relapse, progression, or death was considered an event for PFS estimation. JMP Pro Version 11 software (SAS Institute, Cary, NC, USA) was used for statistical analysis.

## Results

Patient characteristics

A total of 122 patients were identified and further analyzed. There were 74 males (61%), and the median age at diagnosis for the cohort was 64 years (range: 18-98 years). Presenting median counts were as follows: white blood count of 7 x 109/L (normal range: 0.7-183 x 109/L), hemoglobin of 112 g/L (normal range: 67-170), and platelets of 295 x 109/L (normal range: 29-819 x 109/L). A total of 69 patients (57%) were over the age of 60 years. LDH was elevated in 91 (75%) of patients. The median ECOG was 1 (0-4). There were 97 (80%) patients with stage III or IV disease, and 41 (34%) of patients had extra-nodal disease. The median follow-up duration was 32.9 months (range: 0.2-123.7 months) during which a total of 28 (23%) patients experienced disease relapse or progression and 44 (36%) died. The median follow-up of alive patients was 40.2 months. The baseline characteristics of patients are shown in Table 1.

**Table 1:** Baseline characteristics of the cohort ECOG, eastern cooperative oncology group; LDH, lactate dehydrogenase; IPI, International Prognostic Index; R-IPI, revised-IPI; NCCN-IPI, National Comprehensive Cancer Network-IPI; R-CHOP, rituximab, cyclophosphamide, doxorubicin, vincristine, and prednisone; R-CVP, rituximab with cyclophosphamide, vincristine, and prednisone; IFRT, involved field radiotherapy; N/A, not available

Characteristics	N (%)
Age, median (range)	64 (18-98)
Gender, n (%)	
Male	74 (61)
Female	48 (39)
Stage, n (%)	
I/II	25 (20%)
III/IV	97 (80%)
ECOG, median (range)	1 (0-4)
Elevated LDH, n (%)	91 (75%)
Extra-Nodal Disease, n (%)	41 (34%)
IPI, n (%)	
Low	20 (16)
Low-Intermediate	30 (25)
High-Intermediate	32 (26)
High	40 (33)
R-IPI, n (%)	
Very Good	3 (2)
Good	47 (39)
Poor	72 (59)
NCCN-IPI, n (%)	
Low	8 (6)
Low-Intermediate	41 (34)
High-Intermediate	51 (42)
High	22 (18)
Chemotherapy Used, n (%)	
R-CHOP	88 (72%)
R-CHOP/R-CVP	18 (15%)
R-CVP	3 (2%)
Palliative	5 (4%)
Not Treated	8 (7%)
IFRT, n (%)	33 (27%)
End of Treatment Response, n (%)	
Complete Response	76 (62%)
Partial Response	13 (11%)
Progressive Disease	15 (12%)
N/A	18 (15%)
Follow-up months, median (range)	32.9 (0.2-123.7)

Treatment received and response assessment

Standard frontline therapy was combinational chemotherapy with cyclophosphamide, vincristine, doxorubicin, and prednisone with the monoclonal antibody rituximab (R-CHOP) [9]. This treatment protocol was modified in unfit or elderly patients at the discretion of the treating physician to reduce or omit doxorubicin while maintaining a curative approach of therapy. An additional group of patients were deemed unfit for systemic therapy and were given palliative approach to treatment including supportive measures only.

The breakdown of therapy was as follows: the majority of patients received standard R-CHOP at 88 (72%), an additional 18 (15%) received modified R-CHOP, 3 (2%) received R-CVP (rituximab with cyclophosphamide, vincristine, and prednisone), 5 (4%) received palliative rituximab with or without oral chemotherapy, and the remaining 8 (7%) did not receive any systemic therapy and were managed symptomatically. Additionally, 33 (27%) patients received involved field radiotherapy. End of treatment response was as follows: complete response (CR) in 76 (62%), partial response in 13 (11%), progressive disease in 15 (12%), and the remaining 18 (15%) did not have an end of treatment evaluation due to being in the palliative therapy group or early death due to sepsis/multi-organ failure while on active curative intent therapy.

Outcome stratified by IPI

Original IPI

**Figure 1:**
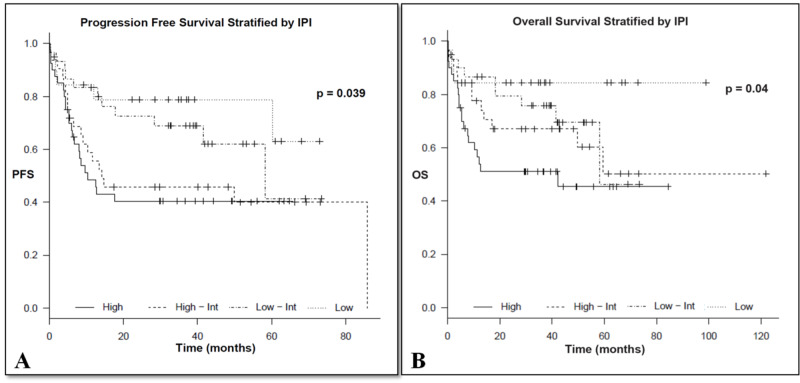
Progression free survival (A) and overall survival (B) stratified by IPI IPI, International Prognostic Index

Stratified according to the different risk groups, a total of 20 (16%), 30 (25%), 32 (26%), and 40 (33%) were scored as low, low-intermediate, high-intermediate, and high, respectively. The corresponding three-year OS (±95% confidence interval) for patients with low, low-intermediate, high-intermediate, and high risk was 84% ± 8, 75.8% ± 8, 67% ± 9 and 51.1% ± 8, respectively (p = 0.04). On the other hand, the three-year PFS (±95% confidence interval) for patients with low, low-intermediate, high-intermediate, and high risk was 78.8% ± 9, 69% ± 9, 45.7% ± 9 and 40.4%, ± 8, respectively (p = 0.039), as shown in Figures 1A, 1B and Table 2.

**Table 2:** Outcome stratified according to different IPI scores IPI, International Prognostic Index; NCCN-IPI, National Comprehensive Cancer Network-IPI

Risk group	Score	Three-year PFS, %	Three-year OS, %
Original IPI			
Low	0-1	78.8% ± 9	84% ± 8
Low-Intermediate	2	69% ± 9	75.8% ± 8
High-Intermediate	3	45.7% ± 9	67% ± 9
High	4-5	40.4% ± 8	51.1% ± 8
Revised IPI			
Very Good	0	100%	100%
Good	1-2	70.8% ± 7	77.6% ± 6
Poor	3-5	42.7% ± 6	58% ± 6
NCCN-IPI			
Low	0-1	83.3% ± 15	100%
Low-Intermediate	2-3	67.4% ± 7	79.6% ± 6
High-Intermediate	4-5	49.6% ± 7	63.5% ± 7
High	6 or above	34.3% ± 11	39.2% ± 11

Revised IPI

**Figure 2:**
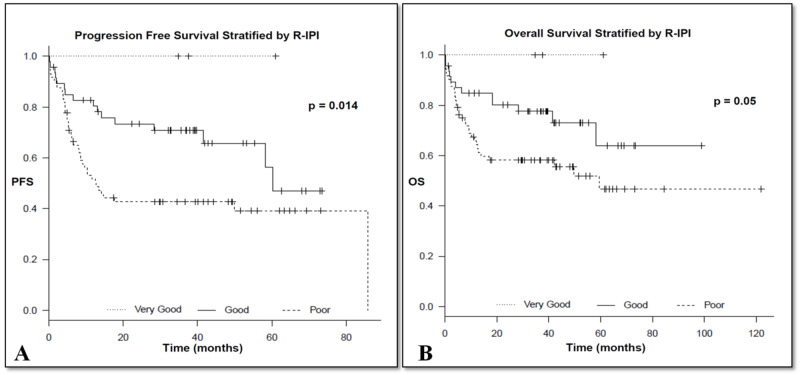
Progression free survival (A) and overall survival (B) stratified by R-IPI R-IPI, revised International Prognostic Index

Stratified according to the different risk groups, a total of 3 (2%), 47 (39%), and 72 (59%) were scored as very good, good, and poor, respectively. The corresponding three-year OS (±95% confidence interval) for patients with very good, good, and poor risk was 100%, 77.6% ± 6, and 58% ± 6, respectively (p = 0.05). On the other hand, the three-year PFS (±95% confidence interval) for patients with very good, good, and poor risk was 100%, 70.8% ± 7 and 42.7% ± 6, respectively (p = 0.014), as shown in Figures 2A, 2B and Table 2.

NCCN-IPI

**Figure 3:**
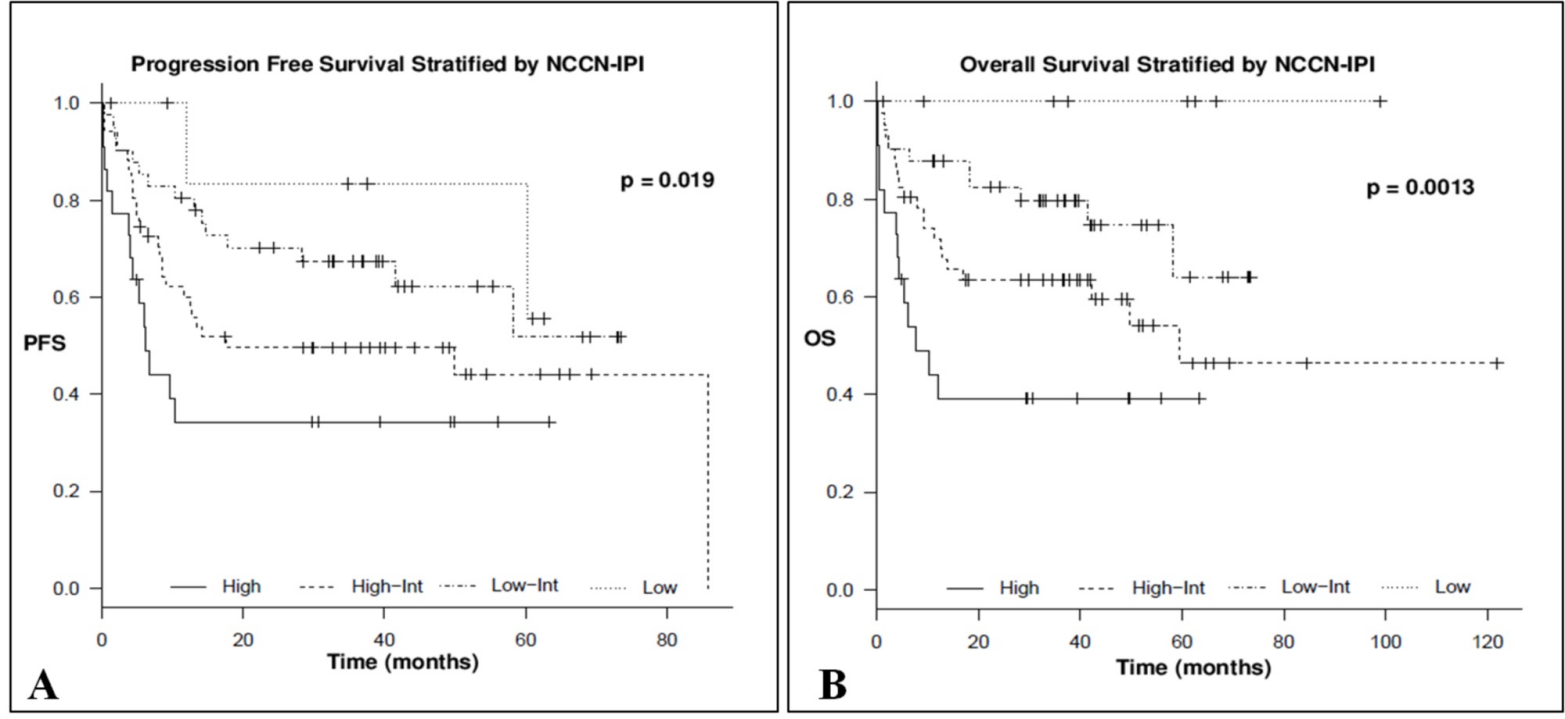
Progression free survival (A) and overall survival (B) stratified by NCCN-IPI NCCN-IPI, National Comprehensive Cancer Network International Prognostic Index

Stratified according to the different risk groups, a total of 8 (6%), 41 (34%), 51 (42%), and 22 (18%) were scored as low, low-intermediate, high-intermediate, and high, respectively. The corresponding three-year OS (±95% confidence interval) for patients with low, low-intermediate, high-intermediate, and high risk was 100%, 79.6% ± 6, 63.5% ± 7, and 39.2% ± 11, respectively (p = 0.0013). On the other hand, the three-year PFS (± 95% confidence interval) for patients with low, low-intermediate, high-intermediate, and high risk was 83.3% ± 15, 67.4% ± 7, 49.6% ± 7, and 34.3% ± 11, respectively (p = 0.019), as shown in Figures 3A, 3B and Table 2.

## Discussion

DLBCL is a common lymphoma representing approximately 25% of all NHL cases. However, it is a heterogeneous disease morphologically, genetically, and even biologically [8]. It is curable in approximately half of the patients, particularly in those who can attain a complete response. The IPI was proposed as a prognostic tool since 1993 to predict OS and RFS in aggressive NHL patients who receive combinational chemotherapy containing doxorubicin. The corresponding five-year OS in the initial group of 2,031 patients assigned to low, low-intermediate, high-intermediate, and high-risk groups was 73%, 51%, 43%, and 26%, respectively. Subsequently, with the incorporation of rituximab as part of combinational chemo-immunotherapy, the original IPI was revised into three risk groups that were initially reported by the British Columbia Cancer Agency and later re-examined using data from three prospective trials [9,10]. Most recently, the NCCN-IPI was derived using the same variables but with further refinement of LDH and age as continuous variables and considering extra-nodal disease involving bone marrow, central nervous system, liver/gastrointestinal tract, or lung as significant. For the four risk groups, i.e., low, low-intermediate, high-intermediate, and high, the resulting five-year OS was 96%, 77%, 56%, and 38%, respectively, and the PFS was 94%, 72%, 54%, and 35%, respectively.

Given the heterogeneous nature of DLBCL, it is unclear whether ethnic background contributes to different disease biology or response to therapy. Furthermore, patients from the MENA region were not represented in the above risk scores. Therefore, the aim of this study is to examine whether the IPI and its subsequent derivations can be used to predict outcome in patients from the MENA region. We observed that the proportion of patients with low/very good risk disease is low, which is consistent with prior reports showing a higher percentage of patients in this part of the world presenting with advanced stage or extra-nodal disease [3]. With regard to PFS estimation, although statistically significant, the original IPI did not discriminate well among the four groups specifically between the lower risk groups (low and low-intermediate) and higher risk groups (high-intermediate and high). On the other hand, PFS using R-IPI was more divergent and most significant using the NCCN-IPI. When looking at OS, we again note that the discrimination potential is less profound with the original IPI where the intermediate group (low and high) fare similarly compared with R-IPI and NCCN-IPI. Our findings suggest that the NCCN-IPI is the most useful score to discriminate among the risk groups in MENA patients followed by the R-IPI. This observation was also noted in an Asian cohort of patients where the NCCN-IPI was superior to the original IPI [7].

This analysis is limited by its retrospective nature and sample size. The proportion of patients with low/very good risk disease is lower than previous reports and could be a further limiting factor. Despite these limitations, this report generates additional insight that the NCCN-IPI is the best prognostic tool when evaluating patients from the MENA region. This observation warrants further validation.

## Conclusions

In conclusion, we validated that the IPI and its subsequent iterations were predictive of outcome in DLBCL patients from the MENA region; however, the NCCN-IPI appeared the most prognostic. These results warrant further confirmation.
